# *Candida palmioleophila* candidemia and bacterial co-infection in a 3-month-old infant with biliary atresia

**DOI:** 10.3389/fcimb.2023.1277607

**Published:** 2023-11-02

**Authors:** Shima Aboutalebian, Bahram Nikmanesh, Masoud Mohammadpour, Arezoo Charsizadeh, Hossein Mirhendi

**Affiliations:** ^1^ Department of Parasitology and Mycology, School of Medicine, Isfahan University of Medical Sciences, Isfahan, Iran; ^2^ Mycology Reference Laboratory, Research Core Facilities Laboratory, Isfahan University of Medical Sciences, Isfahan, Iran; ^3^ Department of Medical Laboratory Science, School of Allied Medical Science, Tehran University of Medical Science, Tehran, Iran; ^4^ Zoonoses Research Centre, Tehran University of Medical Sciences, Tehran, Iran; ^5^ Pediatric Intensive Children’s Medical Center, Tehran University of Medical Sciences, Tehran, Iran; ^6^ Immunology, Asthma, and Allergy Research Institute, Tehran University of Medical Sciences, Tehran, Iran

**Keywords:** *Candida palmioleophila*, candidemia, biliary atresia, pediatrics, antifungal susceptibility testing

## Abstract

Candidemia caused by rare and uncommon *Candida* species is becoming more prevalent in pediatric healthcare settings, resulting in significant morbidity and mortality. One such species, *Candida palmioleophila*, is resistant to fluconazole but highly susceptible to echinocandins. Here, we report the first documented case of *C. palmioleophila* candidemia in Iran that occurred in a male infant with biliary atresia who had been hospitalized for 2 months. The patient’s blood and urine cultures were positive for both yeast and bacterial species. Through DNA sequence analysis, the yeast isolate was identified as *C. palmioleophila*. *In vitro* antifungal susceptibility testing of the isolate against amphotericin B, fluconazole, itraconazole, voriconazole, isavuconazole, posaconazole, and nystatin revealed MIC values of 2, 16, 0.25, 0.0625, 0.125, 0.25, and 4 μg/mL, respectively, and minimum effective concentration for caspofungin was 0.031 μg/mL. Despite receiving antibacterial and antifungal therapies, the patient unfortunately expired due to bradycardia and hypoxemia. Proper identification and epidemiological surveillance studies are needed to understand the exact prevalence of these emerging yeast pathogens. Previously reported cases of *C. palmioleophila* infection, primarily associated with bloodstream infections and catheter-related candidemia, were reviewed.

## Introduction

Biliary atresia (BA) is a rare condition that occurs in infants and is characterized by the scarring and blockage of the bile ducts, which prevents the normal flow of bile into the intestine and subsequently causes damage to the liver, leading to scarring, loss of liver tissue and function, and the development of cirrhosis. If left untreated, BA is fatal within the first 2 years of life. The incidence of BA varies among different ethnic groups, with Asian populations being more commonly affected (Hartley et al., 2009). In addition to the complications related to liver damage, BA also predisposes patients to infections and sepsis, such as bacteremia and fungemia. The underlying causes and pathogenesis of BA remain poorly understood (Hartley et al., 2009; Sundaram et al., 2017)

Candidemia is a significant fungal infection in healthcare settings and currently ranks as the third or fourth most common nosocomial bloodstream infection worldwide. It is the most prevalent fungal infection observed in hospitalized children (Pappas et al., 2016). Pediatric patients affected by candidemia face a considerable mortality rate. Among the various *Candida* species implicated in candidemia cases in hospitalized children, *C. parapsilosis* has been consistently reported as the most frequently isolated species in some medical centers (Chan et al., 2015). It is particularly prevalent in infants under 1 year of age and those with catheter-associated candidemia (Puig-Asensio et al., 2014). *C. palmioleophila* has rarely been implicated in invasive infections in humans and animals. Infections caused by this opportunistic pathogen can lead to endogenous endophthalmitis and fungemia associated with intravenous catheters (Sugita et al., 1999; Datta et al., 2015). Interestingly, *C. palmioleophila* displays resistance to fluconazole but exhibits high susceptibility to echinocandins (Jensen and Arendrup, 2011).

This report describes the first documented occurrence of candidemia due to *C. palmioleophila* in Iran. The study involved the isolation of the fungus from a blood sample obtained from a 3-month-old male infant diagnosed with BA. The identification of the isolate was accomplished through DNA sequence analysis. Also, *C. palmioleophila* cases already isolated from patients with bloodstream infections and central venous catheter (CVC)-related candidemia are reviewed.

## Case presentation

On November 18^th^, 2020, a male infant aged 3 months with a history of BA, abdominal distension, jaundice, poor feeding, and suspected sepsis was admitted to the neonatal ICU at Children’s Medical Centre in Tehran, Iran. The infant presented with malaise, pallor, and low blood pressure. During the physical examination, the infant’s blood pressure was measured at 100/90 mm Hg, and the pulse rate was 170 beats per minute. The infant was placed in an oxyhood for respiratory support. Laboratory analysis of the infant’s blood revealed the following results (the numbers in the parentheses are the normal ranges): White blood cell (WBC) count: 24,600 cells/μl (5,000-19,000 cells/μl), hemoglobin level: 10.5 g/dL (9.5-14.1 g/dL), platelet count: 316,000 cells/μl (150,000-450,000 cells/μl), blood sugar level: 196 mg/dL (80-180 mg/dL), sodium level: 135 mmol/L (134-150 mmol/L), potassium level: 3.9 mmol/L (3.5-5.3 mmol/L), magnesium level: 1.4 mg/dL (1.7-2.1 mg/dL), calcium level: 8.8 mg/dL (8.8-10.8 mg/dL), albumin level: 3 g/dL (3-5.3 g/dL), alanine aminotransferase (ALT) level: 161 U/L (30-90 U/L), aspartate aminotransferase (AST) level: 184 U/L (male children: 17-21 U/L), C-reactive protein (CRP) level: 21 mg/L (< 8 mg/L), erythrocyte sedimentation rate (ESR): 57 mm in the 1st hour (>10 mm/hour).

Both blood and urine cultures were positive for *Candida* species and *Pseudomonas* species. The urine culture showed the presence of 50,000 colony-forming units (CFU) of *Candida*, confirming the diagnosis of candiduria.

**Figure 1:**
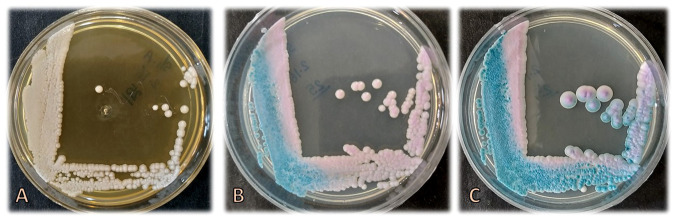
Colonies of *Candida palmioleophila* on Sabouraud dextrose agar after 3 days **(A)** and on CHROMagar *Candida* after 3 **(B)** and 7 **(C)** days of incubation at 35°C.

**Figure 2:**
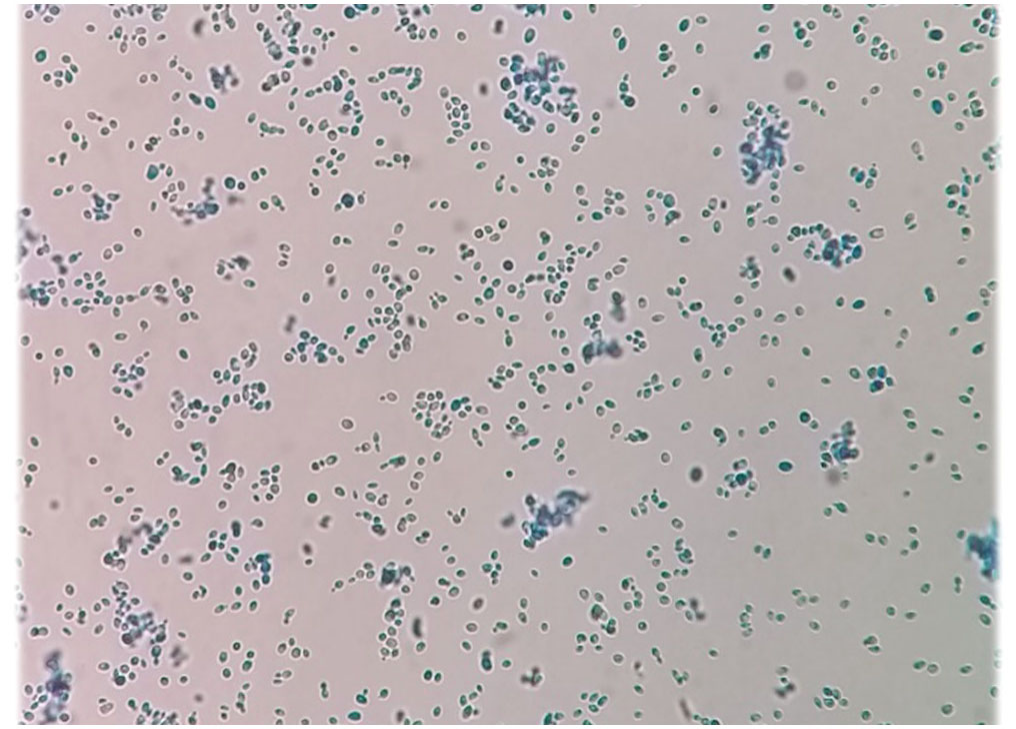
Microscopic examination of the colonies indicating long-oval, multipolar budding yeast cells but no pseudohyphae.

Three ml of the blood sample were inoculated into culture medium bottles (BACTEC Peds Plus/F Culture Vials, Ireland) and processed by an automated blood culture system (Bactec 9120, Becton Dickinson, USA). The system signaled positive for the blood sample, and then 100 μl of the bottle sample was streaked on MacConkey agar, blood agar, chocolate agar (Merck, Germany), and Sabouraud dextrose agar (SDA) plates containing chloramphenicol (50 mg/L) (Biolife, Italy) (**Figure 1**), were incubated at 35°C and examined daily. Additionally, yeast colonies grown on SDA were subcultured on CHROMagar *Candida* (CHROMagar Paris, France) to purify the isolate and preliminary species identification. After 3 days (**Figure 1**) and 7 days (**Figure 1**), rose quartz to turquoise-colored, smooth, slightly shiny colonies were yielded. Direct microscopic examination of the colonies showed long-oval, budding multipolar cells yeast but was unable to produce pseudohyphae (**Figure 2**). The isolate was tested for germ tube production, which was negative within 24 h; these results confirmed the presence of a non-*albicans Candida* species.

After 5 days of hospitalization, culturing drain fluids were positive for *Acinetobacter* sp.; therefore, broad-spectrum antibacterial therapy (colistin, ceftazidime, vancomycin, and amikacin) and antifungal therapy (amphotericin B for 10 days) were initiated. In addition, magnesium sulfate was prescribed due to low magnesium. However, the patient’s symptoms did not improve. On day 50, the patient was transferred to the ICU due to hypoxia, malaise, weakness, fever, bloody stools, and unstable clinical conditions. Blood cultures were again positive for *Candida* sp. and *Pseudomonas* sp. Urine culture was also positive for *Klebsiella* sp. CVC was removed, and treatment was switched to fluconazole, cefepime, cotrimoxazole, and meropenem.

During hospitalization, the patient developed abdominal distension and hypoglycemia. On day 54, laparotomy was performed to examine the abdominal organs because several bands had caused adhesions and obstruction of the ileum and jejunum, which was released, and then the colon was internalized. Abdominal ultrasound implied the presence of BA. There was neither free gas nor fluid in the abdomen or pelvis and no evidence of other intestinal obstruction. Despite antibacterial and antifungal therapy, the patient’s situation deteriorated, and the patient died on day 59 due to bradycardia and hypoxemia.

The yeast isolated from blood was sent to the medical mycology laboratory, School of Medicine, Isfahan University of Medical Sciences (IUMS), Isfahan, Iran, for identification and antifungal susceptibility testing. Total DNA was extracted by the boiling method (Salehipour et al., 2021), ITS1-5.8-ITS4 region of rDNA was amplified (Aboutalebian et al., 2022a), purified, and sequenced at Core Facilities Laboratory, IUMS, using BigDye^®^ Terminator Cycle Sequencing (Applied Biosystems) with the primer ITS1. A BLAST algorithm search revealed 99.83% identity of the sequence (only one adenine (A) deletion) with *Candida palmioleophila.* The sequence obtained was deposited in GenBank under accession number OR431745.

In vitro antifungal susceptibility testing (AFST) was performed based on the CLSI M27-A2 protocol. The concentrations of amphotericin B (AMB), itraconazole (ITC), voriconazole (VRC), posaconazole (POS), isavuconazole (ISA) and nystatin (NYS) ranged from 0.031 to 16 μg/mL; the fluconazole (FLC) assay range was 0.125 to 64 μg/mL; and caspofungin (CAS) ranged from 0.016 to 8 μg/mL. AFST of the isolate against AMB, FLC, ITC, VRC, POS, ISA, and NYS revealed MIC values of 2, 16, 0.25, 0.0625, 0.25, 0.125, and 4 μg/mL, respectively, and minimum effective concentration (MEC) for CAS was 0.031 μg/mL. *Candida parapsilosis* ATCC 22019 and *Candida krusei* ATCC 6258 were included in the tests run as the quality controls.

## Discussion

Invasive candidiasis is a severe condition with a wide range of clinical presentations that can potentially affect any organ, posing a lethal risk to critically ill children (Zaoutis, 2010). Independent risk factors associated with candidemia-related mortality in neonates include prematurity, low birth weight, prolonged hospitalization (particularly in neonatal and pediatric intensive care units), extended use of broad-spectrum antibiotics, presence of central venous catheters (CVC), utilization of total parenteral nutrition, treatment with mechanical ventilation, colonization of skin and mucosal surfaces, and immunodeficiency (Aboutalebian et al., 2022b). The most common causative agents of candidemia in pediatric and adult patients are *C. parapsilosis*, *C. albicans*, *C. krusei*, *C. glabrata*, and *C. tropicalis*, accounting for over 90% of all reported cases (Pappas et al., 2016). However, recent epidemiological studies have highlighted an emerging trend of increasing incidence of candidemia caused by less common species.

The case report presented here describes the medical history of a male infant diagnosed with BA. Laboratory analysis revealed an elevated white blood cell count, and both blood and urine cultures showed positive results for *Candida* species, confirming the presence of candidemia and candiduria. Further molecular testing identified the specific *Candida* isolate as *C. palmioleophila*. Antifungal ssusceptibility testing indicated that the isolate had lower MICs to itraconazole, voriconazole, isavuconazole, and caspofungin, compared with amphotericin B, fluconazole, posaconazole, and nystatin that had higher MICs. These results are in line with the report of Arendrup et al. (2011).

Unfortunately, despite medical efforts, the infant ultimately succumbed to hypoxemia and bradycardia, resulting in his demise.

*C. palmioleophila* is frequently misidentified as *C. famata* (*Debaryomyces hansenii*) or *C. guilliermondii* (*Pichia guilliermondii*). Unlike species complexes such as *C. albicans*, *C. parapsilosis*, and *C. glabrata*, accurate identification of the *C. guilliermondii* and *C. famata* groups is vital for effective clinical management, as these species exhibit varying susceptibility profiles (Jensen and Arendrup, 2011). It is crucial to avoid misidentification of these fungal species as it can undermine the accuracy of epidemiological studies and compromise antibiotic susceptibility assessments (Aboutalebian et al., 2023), potentially leading to an inaccurate understanding of the prevalence of these species.

The increasing prevalence of non-*albicans Candida* species, including *C. palmioleophila*, has raised concerns due to their distinct traits and resistance to fluconazole. Like *C. auris*, echinocandins are the first-line treatment for *C. palmioleophila* infections (Biagi et al., 2019). Several molecular methods have been proposed for the accurate identification of *C. palmioleophila*, including probe-based techniques (Mota et al., 2012), PCR-restriction fragment length polymorphism (RFLP) (Feng et al., 2014), and multiplex PCR with species-specific primers (Feng et al., 2014). Recognizing the importance of precise identification to avoid inappropriate treatment and adverse outcomes, we employed the sequencing of the ITS region of rDNA to identify the isolate in question accurately.

This case underscores the significance of early and appropriate antimicrobial therapy in critically ill patients suspected of sepsis. It highlights the challenges in managing invasive fungal infections in such patients and emphasizes the ongoing need for research to develop new, more effective treatment options.

### Review of the literature

*C. palmioleophila* was initially described utilizing molecular techniques in 1988 by Nakase et al. (1988). In 1999, it was reported as an opportunistic pathogen causing fungemia associated with intravenous catheter use in a 34-year-old male with chronic myelogenous leukemia (Sugita et al., 1999). A 6-year national surveillance of fungemia in Denmark reported the identification of nine blood isolates of *C. palmioleophila* between 2007 and 2009; however, no clinical details were recorded for these cases (Arendrup et al., 2011). Additionally, two cases of candidemia induced by *C. palmioleophila* have been documented. One case involved a 54-year-old man who was a drug addict with an infected ulcer in the lower right limb, chronic HCV hepatitis, and lower limb polyneuropathy. This patient was treated using a CVC (Pierantoni et al., 2020). The second case involved a 2-month-old male infant admitted to the neonatal unit with acute respiratory failure due to severe community-acquired bilateral pneumonia. The infant had congenital Clippers Syndrome and pneumothorax and received various antifungal medications such as micafungin, voriconazole, caspofungin, and ambisome. Unfortunately, despite treatment, the patient succumbed to candidemia (Pierantoni et al., 2020). It is worth mentioning that both these cases were initially misidentified as *Candida albicans* using the Vitek 2 system and CHROMagar (Pierantoni et al., 2020). Furthermore, studies on the identification and antifungal susceptibility of yeast strains isolated in Tunisian hospitals have reported two cases of *Candida palmioleophila* infection, although no patient information is available (Eddouzi et al., 2013). Another reported case involves a 51-year-old man with acute myeloid leukemia, Pneumococcus pneumonia, and endogenous fungal endophthalmitis (Datta et al., 2015).

Kyriakidis et al. (2019) documented a single case of *C. albicans* candidemia associated with BA, in a 31-day-old female infant of Nigerian descent. This particular case involved the presence of extrahepatic BA and double heterozygosity for sickle cell disease and alpha-thalassemia. The infant effectively received treatment by administering neomycin and liposomal amphotericin B (Kyriakidis et al., 2019).

## Conclusion

We presented a case of candidemia caused by infection with *Candida palmioleophila*, highlighting its emergence as a potential pathogen in newborns with BA. The correct identification of this pathogen is crucial due to its distinctive susceptibility profile. We recommend conducting molecular epidemiological surveillance studies to assess the prevalence of these emerging pathogens.

## Author contributions

SA: Conceptualization, Data curation, Formal Analysis, Investigation, Methodology, Resources, Software, Validation, Visualization, Writing – original draft, Writing – review & editing. BN: Investigation, Writing – review & editing. MM: Investigation, Writing – review & editing. AC: Investigation, Writing – review & editing. HM: Conceptualization, Funding acquisition, Project administration, Supervision, Validation, Visualization, Writing – review & editing.

## References

[B14] Aboutalebian S. Charsizadeh A. Eshaghi H. Nikmaesh B. Mirhendi H. (2023). A case of Candida metapsilosis conjunctivitis in a neonate admitted to the cardiac heart intensive care unit. Clin. Case Rep. 11 (1), e6870. doi: 10.1002/ccr3.6870 36703771 PMC9869643

[B10] Aboutalebian S. Mahmoudi S. Charsizadeh A. Nikmanesh B. Hosseini M. Mirhendi H. (2022a). Multiplex size marker (YEAST PLEX) for rapid and accurate identification of pathogenic yeasts. J. Clin. Lab. Anal., e24370. doi: 10.1002/jcla.24370 35318737 PMC9102616

[B12] Aboutalebian S. Mirhendi H. Eshaghi H. Nikmaesh B. Charsizadeh A. (2022b). The first case of Wickerhamomyces anomalus fungemia in Iran in an immuneodeficient child, a review on the literature. J. Med. Mycology, 101351. doi: 10.1016/j.mycmed.2022.101351 36413850

[B13] Arendrup M. C. Bruun B. Christensen J. J. Fuursted K. Johansen H. K. Kjaeldgaard P.. (2011). National surveillance of fungemia in Denmark (2004 to 2009). J. Clin. Microbiol. 49 (1), 325–334. doi: 10.1128/JCM.01811-10 20980569 PMC3020479

[B15] Biagi M. J. Wiederhold N. P. Gibas C. Wickes B. L. Lozano V. Bleasdale S. C. (Eds.) (2019). Development of high-level echinocandin resistance in a patient with recurrent Candida auris candidemia secondary to chronic candiduria. Open forum infectious diseases (US: Oxford University Press US) 6 (7), ofz262. doi: 10.1093/ofid/ofz262 PMC6602379 31281859

[B4] Chan S. Baley E. D. Hossain J. Di Pentima M. C. (2015). Candida species bloodstream infections in hospitalised children: a 10-year experience. J. paediatrics Child Health 51 (9), 857–861. doi: 10.1111/jpc.12905 PMC5646212 25941056

[B7] Datta N. Arendrup M. C. Saunte J. P. (2015). First report of Candida palmioleophila endogenous endophthalmitis. Acta ophthalmologica 93 (6), e517–e5e8. doi: 10.1111/aos.12662 25626724

[B20] Eddouzi J. Lohberger A. Vogne C. Manai M. Sanglard D. (2013). Identification and antifungal susceptibility of a large collection of yeast strains isolated in Tunisian hospitals. Med. mycology 51 (7), 737–746. doi: 10.3109/13693786.2013.800239 23768242

[B17] Feng X. Wu J. Ling B. Yang X. Liao W. Pan W.. (2014). Development of two molecular approaches for differentiation of clinically relevant yeast species closely related to Candida guilliermondii and Candida famata. J. Clin. Microbiol. 52 (9), 3190–3195. doi: 10.1128/JCM.01297-14 24951804 PMC4313136

[B1] Hartley J. L. Davenport M. Kelly D. A. (2009). Biliary atresia. Lancet 374 (9702), 1704–1713. doi: 10.1016/S0140-6736(09)60946-6 19914515

[B8] Jensen R. H. Arendrup M. C. (2011). Candida palmioleophila: characterization of a previously overlooked pathogen and its unique susceptibility profile in comparison with five related species. J. Clin. Microbiol. 49 (2), 549–556. doi: 10.1128/JCM.02071-10 21147953 PMC3043495

[B21] Kyriakidis I. Palabougiouki M. Vasileiou E. Tragiannidis A. Stamou M. Moudiou T.. (2019). Candidemia complicating biliary atresia in an infant with hemoglobinopathy. Turk pediatri arsivi 54 (2), 129–132. doi: 10.14744/TurkPediatriArs.2019.67674 31384149 PMC6666363

[B16] Mota A. J. Back-Brito G. N. Nobrega F. G. (2012). Molecular identification of Pichia guilliermondii, Debaryomyces hansenii and Candida palmioleophila. Genet. Mol. Biol. 35, 122–125. doi: 10.1590/S1415-47572011005000059 22481884 PMC3313500

[B18] Nakase T. Itoh M. Suzuki M. Komagata K. Kodama T. (1988). Candida palmioleophila sp. nov., a yeast capable of assimilating crude palm oil, formerly identified as Torulopsis candida. J. Gen. Appl. Microbiol. 34 (6), 493–498. doi: 10.2323/jgam.34.493

[B3] Pappas P. G. Kauffman C. A. Andes D. R. Clancy C. J. Marr K. A. Ostrosky-Zeichner L.. (2016). Clinical practice guideline for the management of candidiasis: 2016 update by the Infectious Diseases Society of America. Clin. Infect. Dis. 62 (4), e1–e50. doi: 10.1093/cid/civ933 26679628 PMC4725385

[B19] Pierantoni D. C. Bernardo M. Mallardo E. Carannante N. Attanasio V. Corte L.. (2020). Candida palmioleophila isolation in Italy from two cases of systemic infection, after a CHROMagar and Vitek system mis-identification as C. albicans. New microbiologica 43 (1), 47–50. 31814032

[B5] Puig-Asensio M. Padilla B. Garnacho-Montero J. Zaragoza O. Aguado J. Zaragoza R.. (2014). Epidemiology and predictive factors for early and late mortality in Candida bloodstream infections: a population-based surveillance in Spain. Clin. Microbiol. Infection 20 (4), O245–OO54. doi: 10.1111/1469-0691.12380 24125548

[B9] Salehipour K. Aboutalebian S. Charsizadeh A. Ahmadi B. Mirhendi H. (2021). Differentiation of Candida albicans complex species isolated from invasive and non-invasive infections using HWP1 gene size polymorphism. Curr. Med. mycology 7 (2), 34. doi: 10.18502/cmm.7.2.7034 PMC8740857 35028483

[B6] Sugita T. Kagaya K. Takashima M. Suzuki M. Fukazawa Y. Nakase T. (1999). A clinical isolate of Candida palmioleophila formerly identified as Torulopsis candida. Nippon Ishinkin Gakkai Zasshi 40 (1), 21–25. doi: 10.3314/jjmm.40.21 9929578

[B2] Sundaram S. S. Mack C. L. Feldman A. G. Sokol R. J. (2017). Biliary atresia: Indications and timing of liver transplantation and optimization of pretransplant care. Liver Transplant. 23 (1), 96–109. doi: 10.1002/lt.24640 PMC5177506 27650268

[B11] Zaoutis T. (2010). Candidemia in children. Curr. Med. Res. Opin. 26 (7), 1761–1768. doi: 10.1185/03007995.2010.487796 20513207

